# Prognosis analysis of necroptosis-related genes in colorectal cancer based on bioinformatic analysis

**DOI:** 10.3389/fgene.2022.955424

**Published:** 2022-08-15

**Authors:** Xiaojie Liang, Zhaoxiang Cheng, Xinhao Chen, Jun Li

**Affiliations:** ^1^ Department of General Surgery, The Affiliated Jiangning Hospital of Nanjing Medical University, Nanjing, China; ^2^ Department of General Surgery, Jiangning Traditional Chinese Medicine Hospital, Nanjing, China; ^3^ Department of Hepatobiliary and Pancreatic Surgery, The Affiliated Jiangning Hospital of Nanjing Medical University, Nanjing, China

**Keywords:** colorectal cancer, necroptosis-related genes, prognostic signature, bioinformatics analysis, TCGA, GEO

## Abstract

**Background:** Colorectal cancer (CRC) is one gastrointestinal malignancy, accounting for 10% of cancer diagnoses and cancer-related deaths worldwide each year. Therefore, it is urgent to identify genes involved in CRC predicting the prognosis.

**Methods:** CRC’s data were acquired from the Gene Expression Omnibus (GEO) database (GSE39582 and GSE41258 datasets) and The Cancer Genome Atlas (TCGA) database. The differentially expressed necroptosis-related genes (DENRGs) were sorted out between tumor and normal tissues. Univariate Cox regression analysis and least absolute shrinkage and selectionator operator (LASSO) analysis were applied to selected DENRGs concerning patients’ overall survival and to construct a prognostic biomarker. The effectiveness of this biomarker was assessed by the Kaplan–Meier curve and the receiver operating characteristic (ROC) analysis. The GSE39582 dataset was utilized as external validation for the prognostic signature. Moreover, using univariate and multivariate Cox regression analyses, independent prognostic factors were identified to construct a prognostic nomogram. Next, signaling pathways regulated by the signature were explored through the gene set enrichment analysis (GSEA). The single sample gene set enrichment analysis (ssGSEA) algorithm and tumor immune dysfunction and exclusion (TIDE) were used to explore immune correlation in the two groups, high-risk and low-risk ones. Finally, prognostic genes’ expression was examined in the GSE41258 dataset.

**Results:** In total, 27 DENRGs were filtered, and a necroptosis-related prognostic signature based on 6 DENRGs was constructed, which may better understand the overall survival (OS) of CRC. The Kaplan–Meier curve manifested the effectiveness of the prognostic signature, and the ROC curve showed the same result. In addition, univariate and multivariate Cox regression analyses revealed that age, pathology T, and risk score were independent prognostic factors, and a nomogram was established. Furthermore, the prognostic signature was most significantly associated with the apoptosis pathway. Meanwhile, 24 immune cells represented significant differences between two groups, like the activated B cell. Furthermore, 32 immune checkpoints, TIDE scores, PD-L1 scores, and T-cell exclusion scores were significantly different between the two groups. Finally, a 6-gene prognostic signature represented different expression levels between tumor and normal samples significantly in the GSE41258 dataset.

**Conclusion:** Our study established a signature including 6 genes and a prognostic nomogram that could significantly assess the prognosis of patients with CRC.

## Introduction

Colorectal cancer (CRC) is widespread as one digestive system malignancy, which is the second and third most common, respectively, in females and males (Arnold et al., 2017; Bray et al., 2018). According to epidemiological studies, colorectal cancer is the result of synergistic effects of environmental, lifestyle, and genetic factors. Among them, gender and increasing age are closely related to the incidence (Syngal et al., 2015). In addition, risk factors such as poor dietary habits, lack of exercise, obesity, and smoking also increase the risk of developing CRC (Brenner et al., 2011). With the promotion of individualized treatment, the treatment options for local and advanced diseases have become more diverse. Current treatments for CRC differ in patients, such as endoscopic local excision, surgical local excision, and downstaging preoperative chemoradiotherapy. Moreover, the overall survival situations of patients in the advanced stage have also been greatly improved with the continuous iteration of treatment technology and the rapid development of treatment methods. However, the incidence and mortality of CRC are still high because most patients develop symptoms at a later stage. On the other hand, the prognosis of patients with metastatic CRC remains poor with certain limitations, despite the progress in clinical diagnostic methods and the application of comprehensive treatment (Song et al., 2015). Therefore, it is crucial to focus on the molecular mechanism of CRC and seek significant biomarkers, which may improve the detection rate of early screening and conduct more targeted individualized treatment.

Necroptosis is one programmed cell necrosis that differs from apoptosis and is independently regulated by aspartic acid. Cells with necroptosis have typical necrotic features such as swelling and cell membrane rupture, and release numerous damage-related molecules, inflammatory cytokines, and chemokines (Galluzzi and Kroemer, 2008). Necroptosis is involved in many pathophysiological processes, such as infection, liver disease, and neurodegenerative diseases (Xie et al., 2020; Dai et al., 2021; Khan et al., 2021). The regulation of necroptosis plays an essential role in immune activities (Lu et al., 2014). Recent research works have revealed that necroptosis also participates in both the pathogenesis and metastasis of tumors (Ji et al., 2020; Yan et al., 2022). Specifically, necroptosis may inhibit tumor proliferation, progression, and metastasis. The combination of necroptosis inducers with immune checkpoint inhibitors may lead to synergistic effects in tumor suppression (Niu et al., 2022). Furthermore, the effect of tumor necrosis on CRC patients may be closely related to systemic and local inflammatory responses (Richards et al., 2012). In addition, necrosis is regarded as an independent prognostic factor when it comes to CRC (Pollheimer et al., 2010). Although the molecular mechanism of necroptosis has been fully studied, its role in tumorigenesis and progression remains to be absolutely clarified. It may probably offer a valuable opinion that pays attention to the signal pathways involved with necroptosis in early diagnosis and targeted therapy of CRC (Meng et al., 2016).

In our study, the sequencing datasets of CRC with the survival information and clinical data were acquired from The Cancer Genome Atlas (TCGA) database (https://xenabrowser.net), and a total of 69 necroptosis-related genes (NRGs) were obtained from the Kyoto Encyclopedia of Genes and Genomes (KEGG) database (https://www.kegg.jp/kegg/) and a previously published article. Then 27 differentially expressed necroptosis-related genes (DENRGs) were acquired by intersecting the differentially expressed genes (DEGs) in CRC with NRGs. Based on the 27 DENRGs, a prognostic signature was constructed and then determined through a series of statistical analyses. Six significant biomarkers were acquired and then verified in the GSE41258 dataset. Correlations between risk scores and different clinical characteristics were also analyzed. Gene Ontology (GO) and KEGG enrichment analyses were implemented on the two different risk groups using gene set enrichment analysis (GSEA) software, and the immune infiltration analysis was performed using the single sample gene set enrichment analysis (ssGSEA) algorithm.

Immune checkpoints, tumor immune dysfunction and exclusion (TIDE) score, PD-L1 treatment score, T-cell rejection score, and T-cell dysfunction score were also analyzed in both groups. Drug sensitivity analysis was also implemented using the pRRophetic algorithm. Through the aforementioned analysis, we depicted the role of necroptosis in CRC from different aspects. Our study may provide a much more comprehensive theory for further studies.

## Materials and methods

### Data source

Data regarding colon adenocarcinoma (COAD), as well as rectum adenocarcinoma (READ), were obtained from the TCGA database. The TCGA-COAD and READ datasets included 616 tumor samples and 51 normal samples, of which 584 tumor samples contained clinical information and were regarded as a training set for the construction of prognostic signatures. Furthermore, the GSE39582 and GSE41258 datasets related to CRC were obtained from the Gene Expression Omnibus (GEO) database. The GSE39582 dataset, containing 562 tumor samples with survival information, was defined as an external validation set for confirming the prognostic signature. The GSE41258 dataset, containing 186 tumor and 54 normal samples, was used to further examine the expression of signature genes. Furthermore, we downloaded NRGs from the KEGG database; after merging them with NRGs collected from a previous study (Wang and Liu, 2021) and removing duplicate genes, a total of 69 NRGs were obtained (Supplementary Table S1).

### Identification of DEGs and DENRGs

The DEGs were identified between 661 tumor samples and 51 normal ones from the TCGA database using the limma package of R software. Adjusted *p*-value < 0.05 and |log2fold change (FC)| > 0.5 were set as the cutoff values. Next, the DENRGs were screened *via* overlapping the DEGs and NRGs by Venn tool.

### Development and validation of the necroptosis-related signature

Univariate Cox analysis was first used to select prognostic-related genes with *p*-value < 0.2 in the training set. Afterward, the results obtained earlier were incorporated into the least absolute shrinkage and selectionator operator (LASSO) algorithm (Tibshirani, 1997) and were used to screen necroptosis-related prognostic signature genes using the glmnet R package (Engebretsen and Bohlin, 2019). Furthermore, the individualized risk scores of each CRC patient were calculated by the following formula:

Risk Score=Σi Coefficient(genei)∗Exp(genei) (1)

According to the median of the risk scores, the patients in the training set were categorized into two different risk groups. The differences in the overall survival (OS) between the two groups were compared by the Kaplan–Meier method and log-rank test. In addition, receiver operating characteristic (ROC) curves were plotted to assess the efficiency of the model using the survivalROC package (Xie et al., 2012). Similarly, these aforementioned methods were used to validate the performance of the necroptosis-related prognostic biomarker in the validation set.

### Correlation analysis between risk scores and different clinical characteristics

The wilcox.test function was applied to explore the correlations between risk scores and different clinical characteristics in the training set. The variables included in the wilcox.test analysis included age, gender, pathologic T, pathologic N, pathologic M, cancer status, and tumor stage. Next, univariate and multivariate Cox regression analyses were used to select the independent prognostic factors from the risk score and different clinical characteristics. Finally, a nomogram was drawn using the R package RMS on the basis of multivariate Cox regression analyses. A calibration curve and decision curve (DCA) of the nomogram were used to assess the performance of the nomogram on the basis of the prognostic signature.

### Functional enrichment analysis

The GO biological process and KEGG pathways concerning the prognostic signature were explored based on all genes in the two different risk groups in the training set by GSEA v 4.0.3 (Suárez-Fariñas et al., 2010). At the same time, c2.cp.kegg.v7.4.symbols.gmt was selected as the reference gene set, and a NOM *p*-value < 0.05 was regarded as significant enrichment.

### Immune microenvironment analysis

The ssGSEA algorithm was used to calculate the 28 immune cell infiltration abundances of CRC patients in high- and low-risk groups from the training set. Moreover, the expressions of immune checkpoints between the two subgroups were extracted. TIDE was used to evaluate the TIDE score, PD-L1 score, T-cell exclusion score, and T-cell dysfunction score of two subgroups.

### Drug sensitivity analysis based on GDSC

According to the TCGA gene expression profile and the cell line expression profile in the Genomics of Drug Sensitivity in Cancer (GDSC) database, drug IC50 was predicted using the pRRophetic algorithm (Geeleher et al., 2014).

### Biomarker expression validation

The expressions of the genes mentioned earlier were extracted from expression validation set GSE41258. Moreover, R package ggplot2 (Villanueva and Chen, 2019) was used to draw a boxplot through wilcox.test function to verify the expression of prognostic signature genes.

### Cell Culture

The cell lines used in our study include NCM460, HCT116, and SW480, which were obtained from the China Center for Type Culture Collection (CCTCC). The cells were cultured in Dulbecco’s modified Eagle’s medium (DMEM, Biochannel, China) supplemented with 10% fetal bovine serum (FBS, Gibco, US) and 1% penicillin-streptomycin (Pen Strep, Gibco, US) in a 5% CO_2_-humidified incubator at 37°C. Before the treatment of total RNA isolation, the cells were grown on coverslips in 6-well plates overnight.

### Real-time fluorescence quantitative PCR analysis

The Total RNA was extracted using a RNAeasy™ Animal RNA Isolation Kit with Spin Column (Beyotime, China), and cDNA was synthesized using a HiScript III RT SuperMix for qPCR (+gDNA wiper) (Vazyme, China). The quantitative real-time PCR (qPCR) was performed on a StepOne Plus system (Applied Biosystems, USA) with ChamQ SYBR qPCR Master Mix (Vazyme, China). The primers used to amplify the specific genes are listed as follows:

ACTB (human): forward 5′- CAT​GTA​CGT​TGC​TAT​CCA​GGC -3′, reverse 5′-CTC​CTT​AAT​GTC​ACG​CAC​GAT -3’; CHMP2B (human) forward 5′- GGC​TAT​AAT​CAG​AGA​TCG​AGC​AG -3′, reverse 5′- CTC​GTC​TTC​TGT​TTC​CGT​AGA​TG -3’; CHMP6 (human): forward 5′- GAC​AAG​CTG​AGG​CAG​TAC​CAG​A -3′, reverse 5′- CTG​CTC​CTG​GTA​TCG​CTT​CTT​C -3’; RIPK3 (human): forward 5′- GCT​ACG​ATG​TGG​CGG​TCA​AGA​T -3′, reverse 5′- TTG​GTC​CCA​GTT​CAC​CTT​CTC​G -3’; CXCL1 (human): forward 5′- AGC​TTG​CCT​CAA​TCC​TGC​ATC​C -3′, reverse 5′- TCC​TTC​AGG​AAC​AGC​CAC​CAG​T -3’; GPX4 (human): forward 5′- GAG​GCA​AGA​CCG​AAG​TAA​ACT​AC -3′, reverse 5′- CCG​AAC​TGG​TTA​CAC​GGG​AA -3’; TRAF2 (human): forward 5′- TCC​CTG​GAG​TTG​CTA​CAG​C -3′, reverse 5′- AGG​CGG​AGC​ACA​GGT​ACT​T -3’.

### Statistics

The statistical analysis was implemented with R 4.0.5, and the wilcox.test method was applied to determine whether the differences were significant or not. Student’s *t* test was used in real-time fluorescence quantitative PCR analysis, and the mRNA relative expression was presented as the mean ± SEM. Unless otherwise indicated, a *p*-value < 0.05 was regarded as significant statistically.

## Results

### Identification of DEGs and DENRGs

**FIGURE 1:**
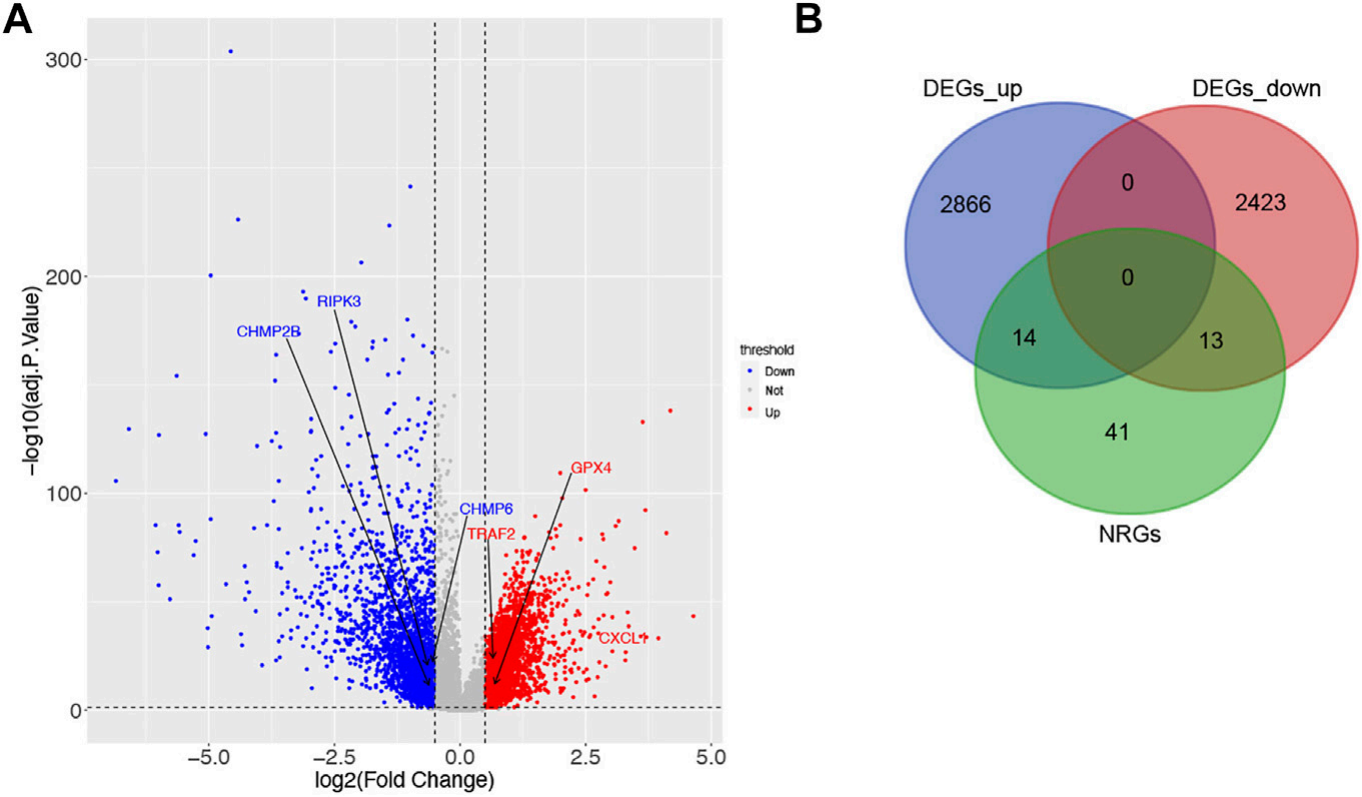
Identification of DEGs and DENRGs in CRC. **(A)** 2880 up-regulated and 2436 down-regulated DEGs based on TCGA. **(B)** 27 DENRGs based on the overlap of DEGs and NRGs.

According to the criteria for DEG, 5316 DEGs (2880 up-regulated and 2436 down-regulated) were selected between 616 tumor samples and 51 normal ones of TCGA (Figure 1A). Next, a total of 27 DENRGs were selected based on overlapping DEGs and NRGs for further analysis (Figure 1B).

### Development of the necroptosis-related signature

**FIGURE 2:**
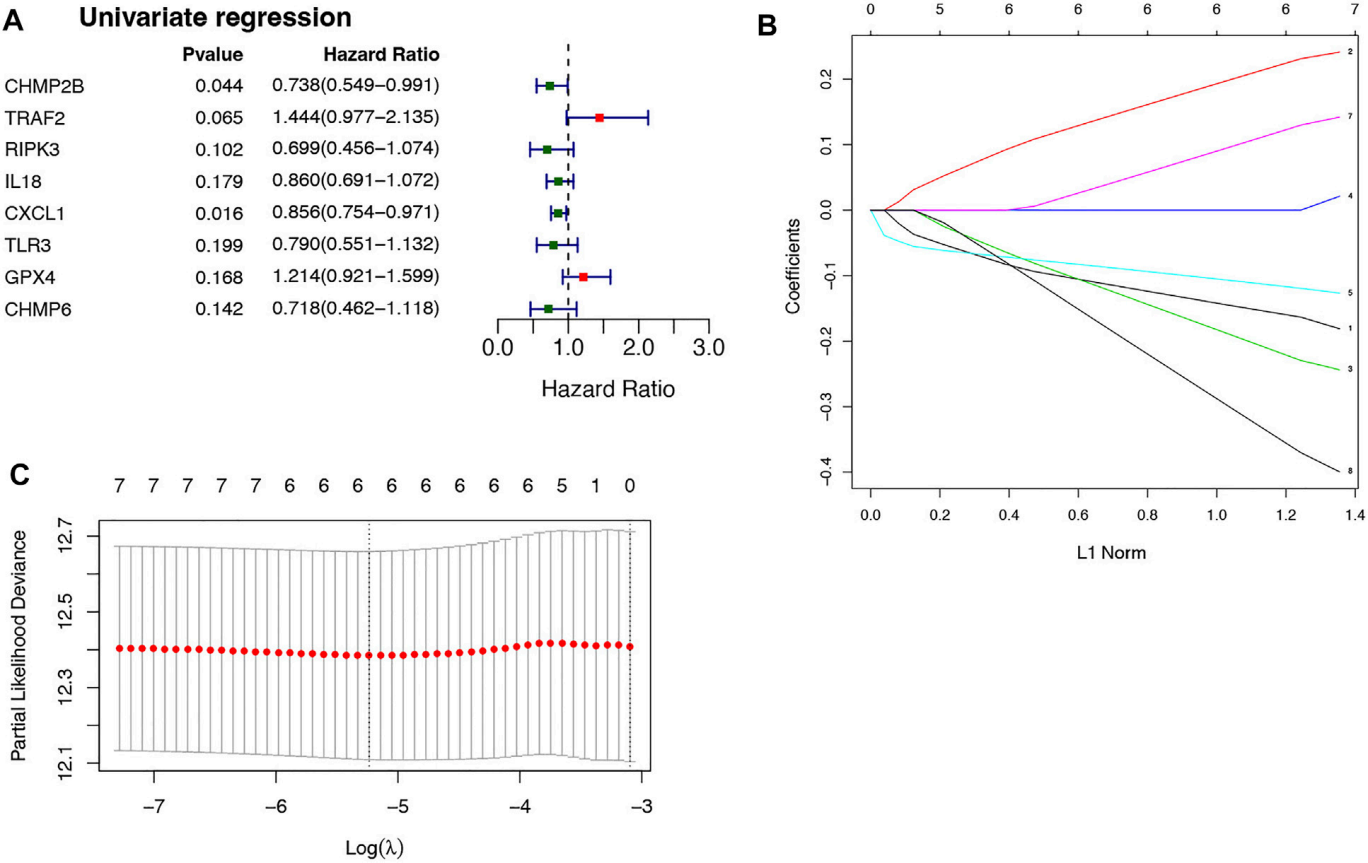
Selection of necroptosis-related genes in CRC. **(A)** 8 DENRGs selected based on the univariate Cox regression analysis. **(B)** The LASSO Cox analysis screened six genes. **(C)** The optimal values of the penalty parameter were defined by 10 fold cross-validation. The two dotted lines represented two particular values of λ. The left side was λmin and the right side was λ1se. The λmin was selected to build the model for accuracy in our study.

Univariate Cox analysis and LASSO regression algorithm were used to select necroptosis-related prognostic signature genes. According to the univariate Cox regression analysis, 8 DENRGs were selected, of which the hazard ratio (HR) of TRAF2 (HR = 1.444, *p* = 0.065, 95% CI = 0.977–2.135) and GPX4 (HR = 1.214, *p* = 0.168, 95% CI = 0.921–1.599) were more than 1, while the HR of the CHMP2B (HR = 0.738, *p* = 0.044, 95% CI = 0.549–0.991), RIPK3 (HR = 0.699, *p* = 0.102, 95% CI = 0.456–1.074), IL18 (HR = 0.860, *p* = 0.179, 95% CI = 0.691–1.072), CXCL1 (HR = 0.856, *p* = 0.016, 95% CI = 0.754–0.971), TLR3 (HR = 0.790, *p* = 0.199, 95% CI = 0.551–1.132), and CHMP6 (HR = 0.718, *p* = 0.142, 95% CI = 0.462–1.118) were less than 1 (Figure 2A). Among 8 DENRGs, 6 DENRGs were screened as necroptosis-related prognostic signature genes, including CHMP2B (lambda = −0.15147441), TRAF2 (lambda = 0.210635396), RIPK3 (lambda = −0.203509488), CXCL1 (lambda = −0.111608948), GPX4 (lambda = 0.108352013), and CHMP6 (lambda = −0.325710435) (Figures 2B,C).

**FIGURE 3:**
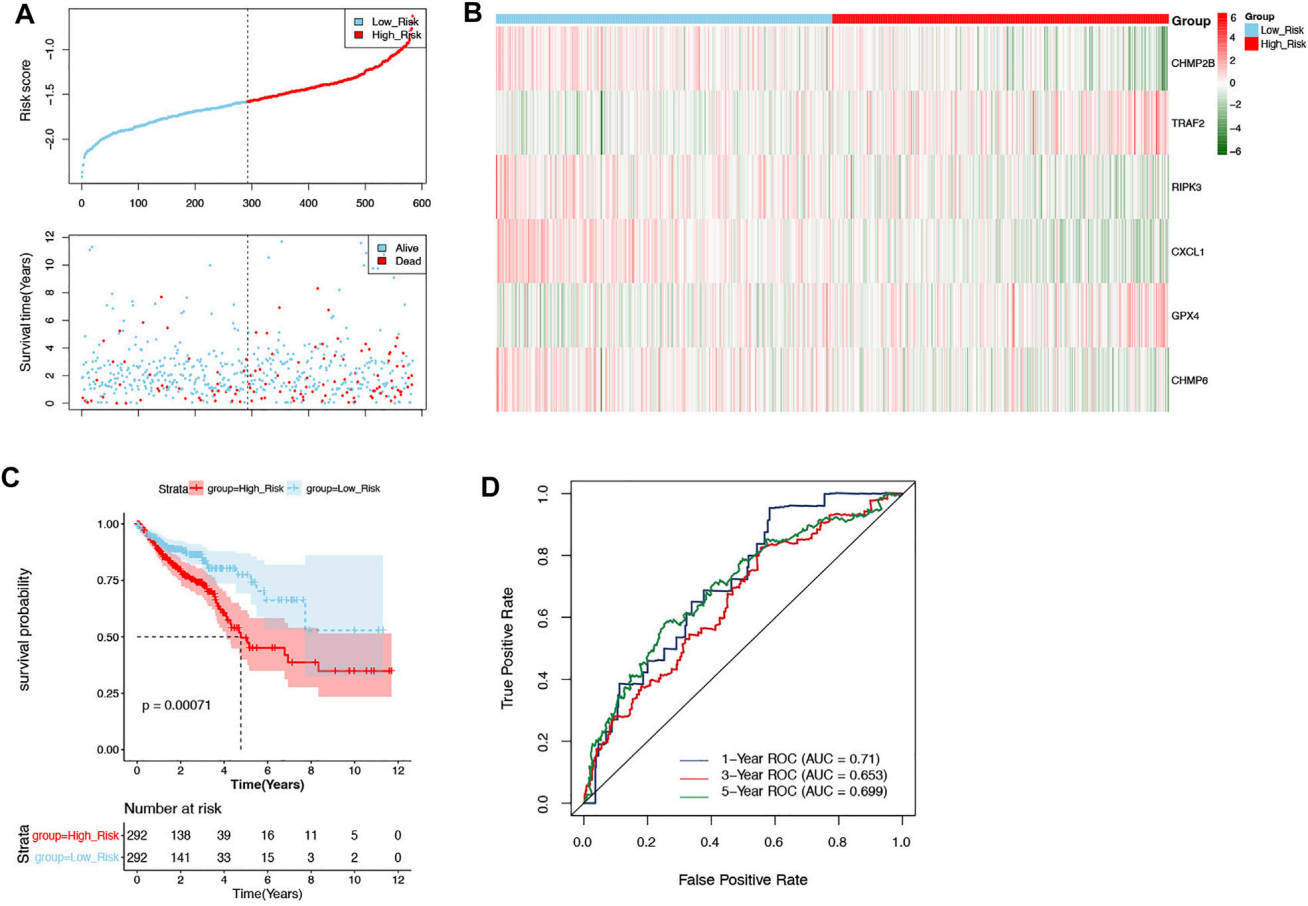
Evaluation of the prognostic signature in the training set. **(A)** The patients in the training set were categorized into high-risk and low-risk groups. **(B)** The expression of 6 DENRGs in high-risk and low-risk groups. **(C)** The Kaplan–Meier curve of high-risk and low-risk groups. **(D)** The ROC curve was used to assess the accuracy of the prognostic signature.

According to the median risk score (−1.578880721), the patients in the training set were divided into two groups: high-risk (*n* = 292) and low-risk groups (*n* = 292) (Figure 3A). As shown in Figure 3B, CHMP2B, RIPK3, CXCL1, and CHMP6 were negatively associated with risk scores, but TRAF2 and GPX4 were positively associated with risk scores. Moreover, the Kaplan–Meier curve displayed that the patients in the high-risk group had a significantly worse OS than those in the low-risk group (Figure 3C, *p* = 0.00071). The ROC curves indicated that the area under the curve (AUC) reached 0.71 at 1 year, 0.653 at 3 years, and 0.699 at 5 years (Figure 3D).

### Validation of the necroptosis-related signature

**FIGURE 4:**
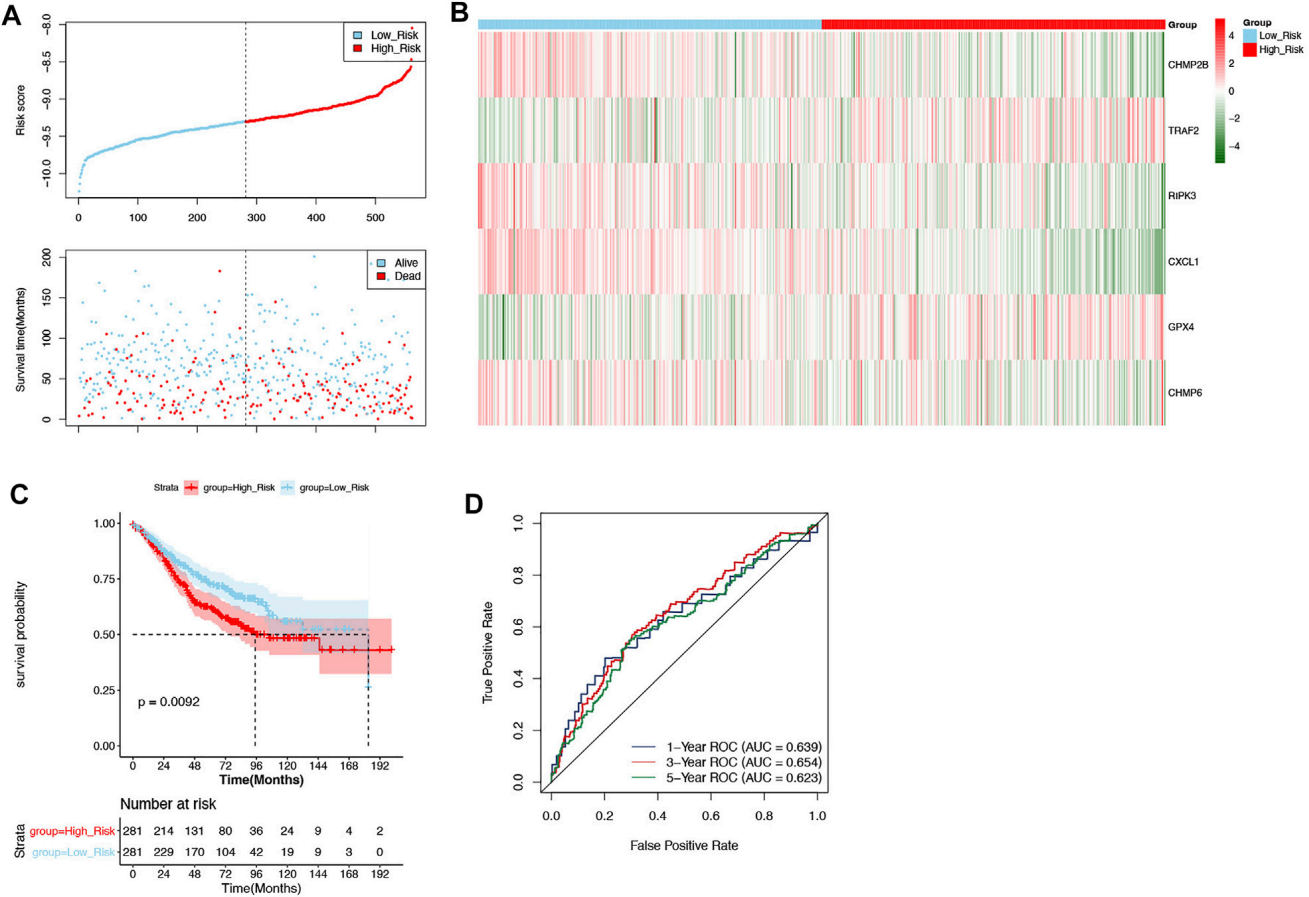
Validation of the prognostic signature in the validation set. **(A)** The patients in the GSE39582 dataset were categorized into high-risk and low-risk groups. **(B)** The expression of 6 DENRGs in high-risk and low-risk groups. **(C)** The Kaplan–Meier curve of high-risk and low-risk groups. **(D)** The ROC curve was used to assess the validity of the prognostic signature.

The GSE39582 dataset was defined as external validation for the prognostic signature, and the patients of this dataset were also divided into two groups: high-risk (*n* = 281) and low-risk (*n* = 281) groups by the median of risk scores (−9.306512262) (Figure 4A). Likewise, the correlation between necroptosis-related prognostic signature genes and risk scores as well as the results of the Kaplan–Meier curve and ROC curves were consistent with those in the training set (Figures 4B–D).

### Correlation analysis between risk scores and different clinical characteristics

**FIGURE 5:**
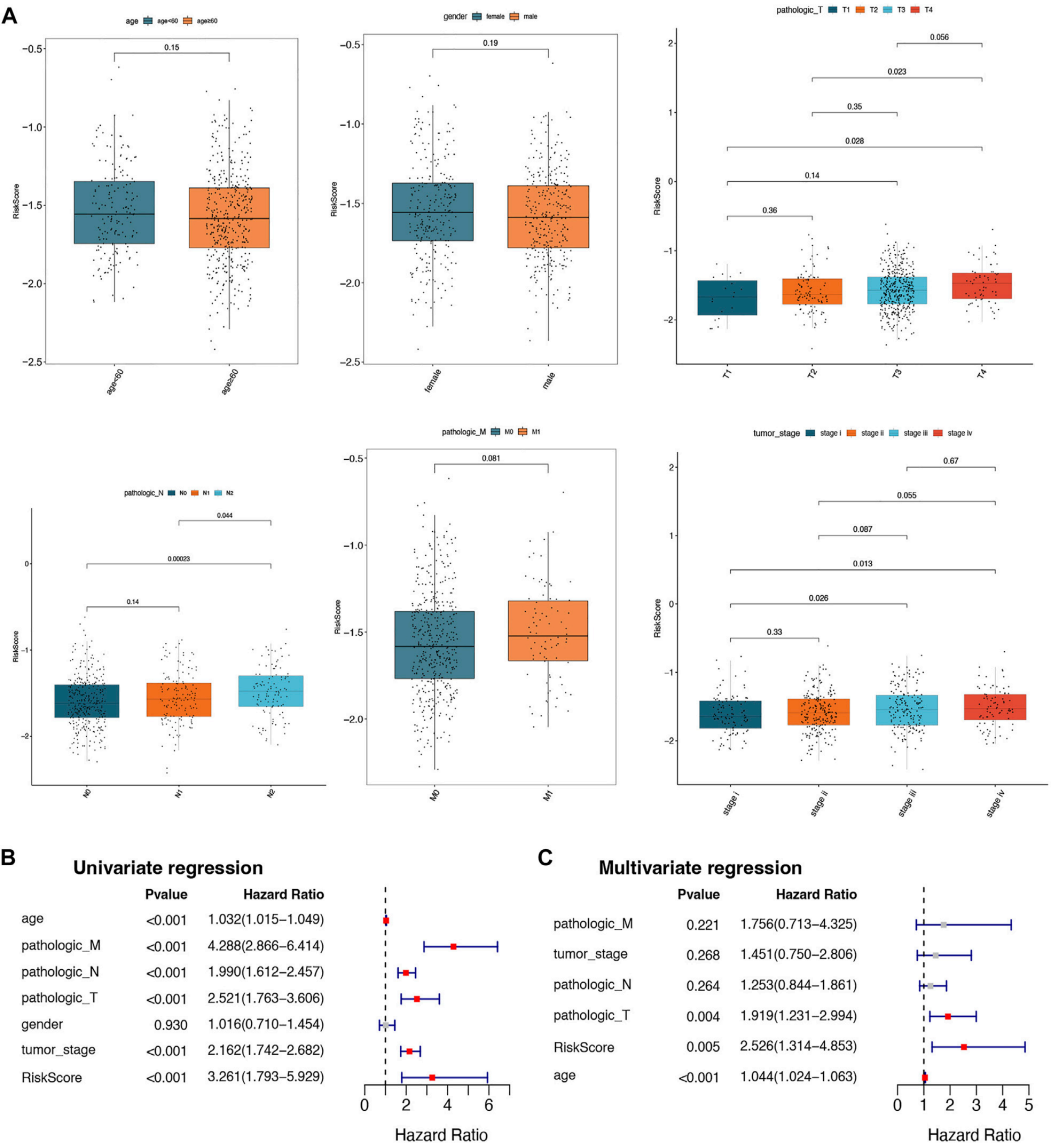
Correlation analysis between risk scores and different clinical characteristics. **(A)** Box plots of correlations between risk score and different characteristics (age, gender, pathologic T, pathologic N, pathologic M, and tumor stage). **(B)** The univariate Cox regression analysis for selected prognostic factors. **(C)** The multivariate Cox regression analysis for selected prognostic factors.

To understand the correlation between the risk score and different clinicopathological features, the differences in the risk scores among the different clinical features (age, gender, pathologic T, pathologic N, pathologic M, and tumor stage) were analyzed in the training set. As depicted in Figure 5A, there were significant differences in risk scores between pathologic N0 and N2, pathologic N1 and N2, pathologic T1 and T4, pathologic T2 and T4, tumor stage I and stage III, and tumor stage I and stage IV, respectively. In addition, univariate and multivariate Cox regression analyses were performed to further investigate the clinicopathological characteristics and prognosis of the prognostic signature. In univariate Cox regression analyses, age; pathologic T, N, and M; tumor stage; and risk score were significantly related to the prognostic signature (*p*-value < 0.05; Figure 5B). The 6 factors with univariate significance were included in the multivariate Cox analysis, indicating that the 3 clinical factors, age, pathology T, and risk score, were independent prognostic factors (*p*-value < 0.05; Figure 5C).

### Establishment of nomograms and calibration curves

**FIGURE 6:**
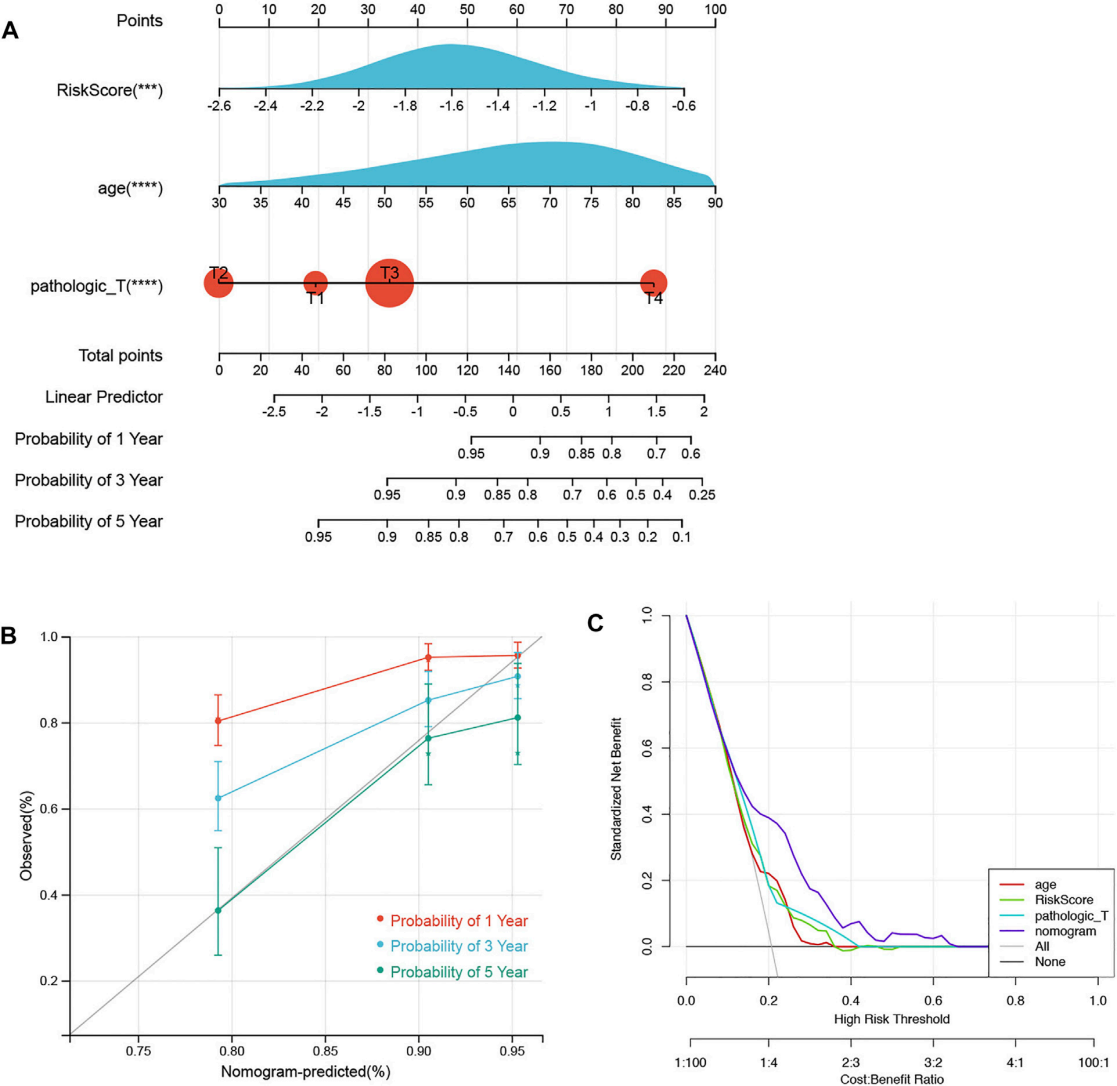
Nomograms and calibration curves. **(A)** Prognostic nomogram was established to predict the OS with 3 independent prognostic factors. **(B)** Calibration curve was used to validate the nomogram based on the prognostic signature. **(C)** DCA was used to validate the nomogram based on the prognostic signature.

In total, 584 patients in the training set were involved in constructing a prognostic nomogram, predicting 1-, 3-, and 5-year OS with 3 independent prognostic factors (age, pathology T, and risk score; Figure 6A). Afterward, the calibration curve and DCA of the nomogram were drawn to validate the nomogram based on the prognostic signature. As shown in Figure 6B, the calibration curve for predicting the 5-year OS indicated that the nomogram-predicted survival closely corresponded with the actual survival outcomes. The net benefit rate of the nomogram model in the DCA curve is higher than the three clinical factors, age, pathological T, and risk score, showing that the nomogram model has an accurate predictive potential with better accuracy for patient prognosis (Figure 6C).

### Functional enrichment analysis

**FIGURE 7:**
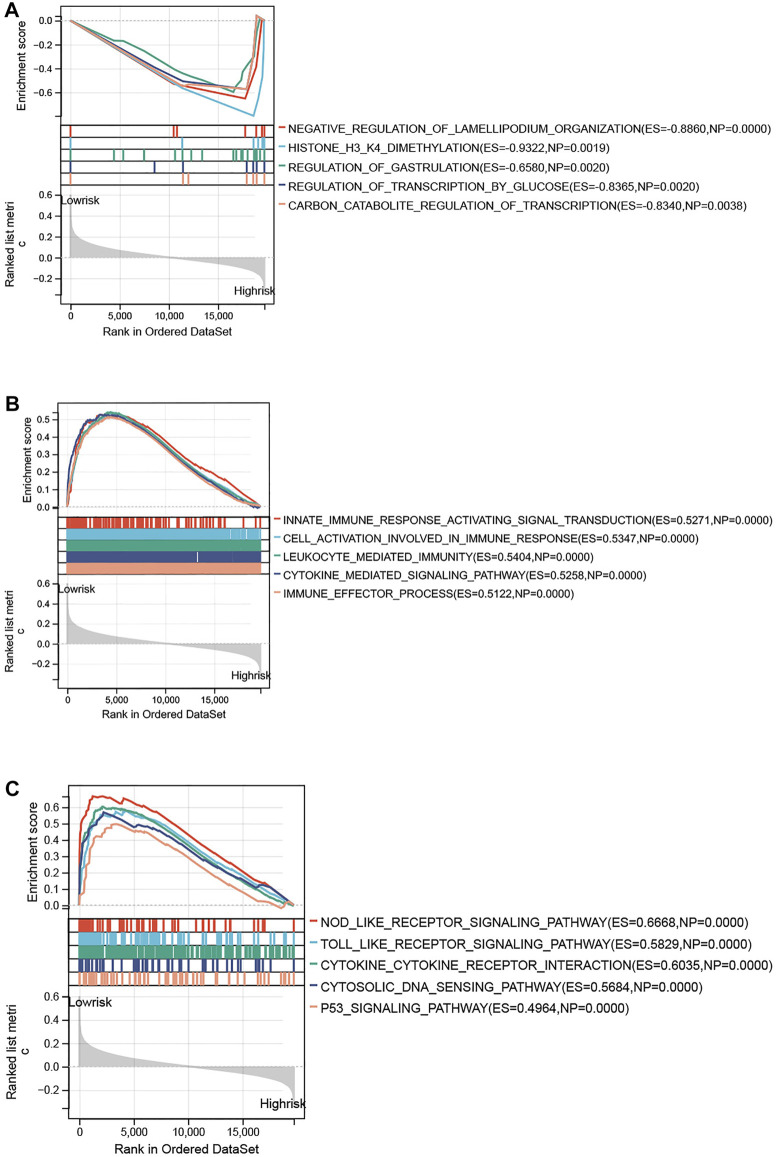
Functional enrichment analysis between high- and low-risk groups. **(A)** Top 5 GO terms enriched in the high-risk group. **(B)** Top 5 GO terms enriched in the low-risk group. **(C)** Top 5 KEGG terms enriched in the low-risk group.

To further explore the specific mechanism of the necroptosis-related signature, GSEA analysis was performed on the training set (low risk vs. high risk). As shown in Figure 7A, the top 5 GO terms were enriched in the high-risk group, including negative regulation of Lamellipodium organization, histone H3 H4 dimethylation, regulation of gastrulation, regulation of transcription by glucose, and carbon catabolite regulation of transcription. The top 5 GO terms were enriched in the low-risk group, including innate immune response activating signal transduction, cell activation involved in immune response, leukocyte-mediated immunity, cytokine-mediated signaling pathway, and immune effector process (Figure 7B). KEGG analysis indicated that the top 5 pathways in the low-risk group were the nod-like receptor signaling pathway, toll-like receptor signaling pathway, cytokine receptor interaction, cytosolic DNA sensing pathway, and p53 signaling pathway (Figure 7C). However, none of the KEGG pathways was enriched in the high-risk group with significance.

### Immune microenvironment analysis

**FIGURE 8:**
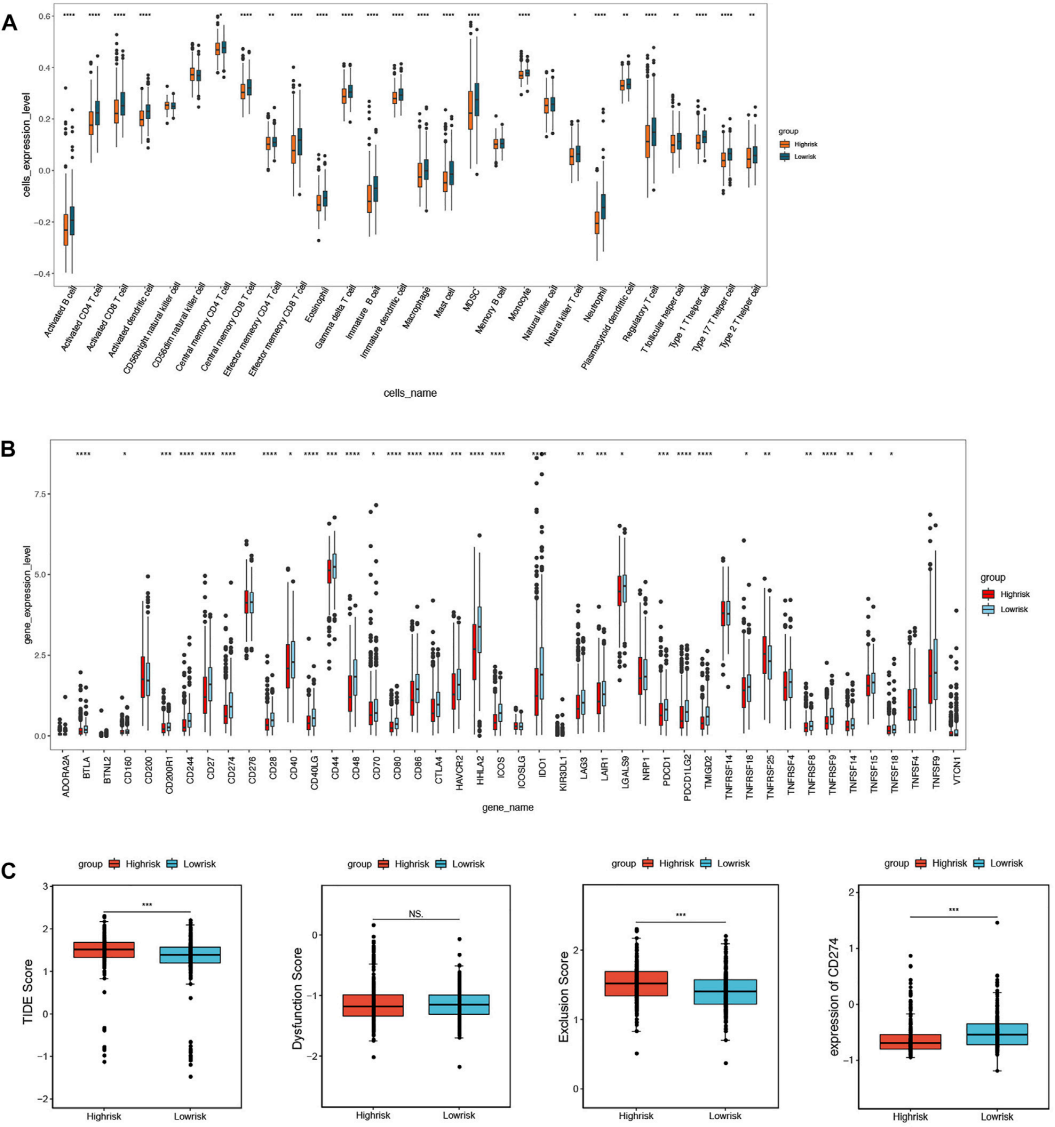
Immune microenvironment analysis between high- and low-risk groups. **(A)** 24 immune cells significantly different based on immune cell infiltration analysis. **(B)** 32 immune checkpoints significantly different based on immune checkpoints analysis. **(C)** TIDE scores, T-cell exclusion scores, and PD-L1 scores significantly different based on TIDE analysis.

The ssGSEA was used to assess immune cell infiltration in the training set; 24 types of immune cells were significantly different between the above groups (*p* < 0.05), including activated B cells, activated CD4 T cells, and activated CD8 T cells (Figure 8A). Furthermore, a total of 32 immune checkpoints represented significant differences as well, such as ADORA2A, BTNL2, and CD160 (Figure 8B). In addition, TIDE analysis demonstrated that the TIDE scores, PD-L1 scores, and T-cell exclusion scores all showed significant differences between the two groups (Figure 8C).

### Drug sensitivity analysis based on GDSC

**FIGURE 9:**
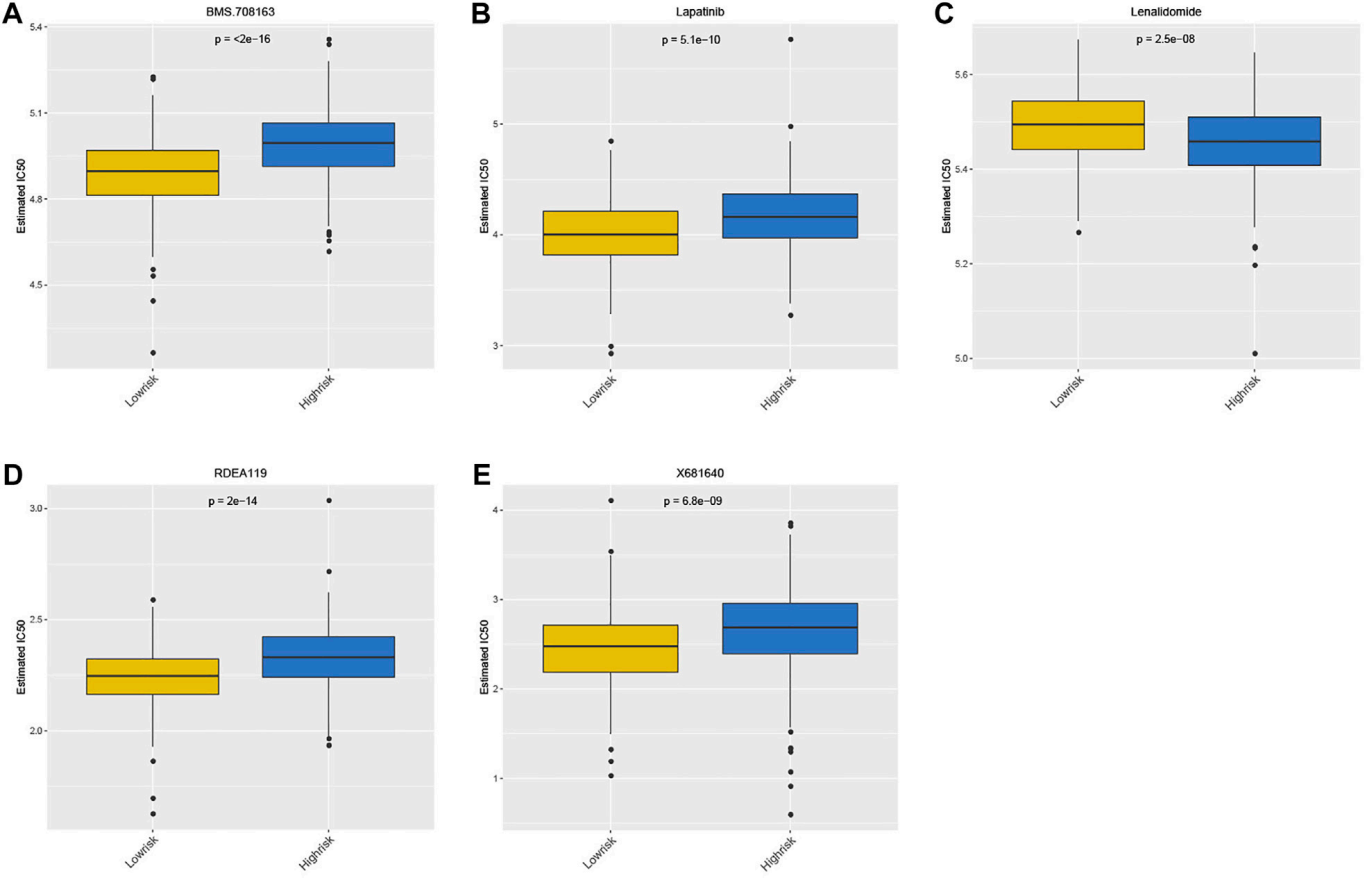
Drug sensitivity analysis between high- and low-risk groups based on GDSC.

The drug sensitivity of patients between the high- and low-risk groups was compared using the GDSC database. Notably, the top 5 drugs shown in Figure 9 indicated that the patients in the high-risk group were more sensitive to BMS.708163, RDEA119, Lapatinib, and X681640. However, the patients in the other group showed more sensitivity to lenalidomide (Figure 9).

### Validation of biomarker expression

**FIGURE 10:**
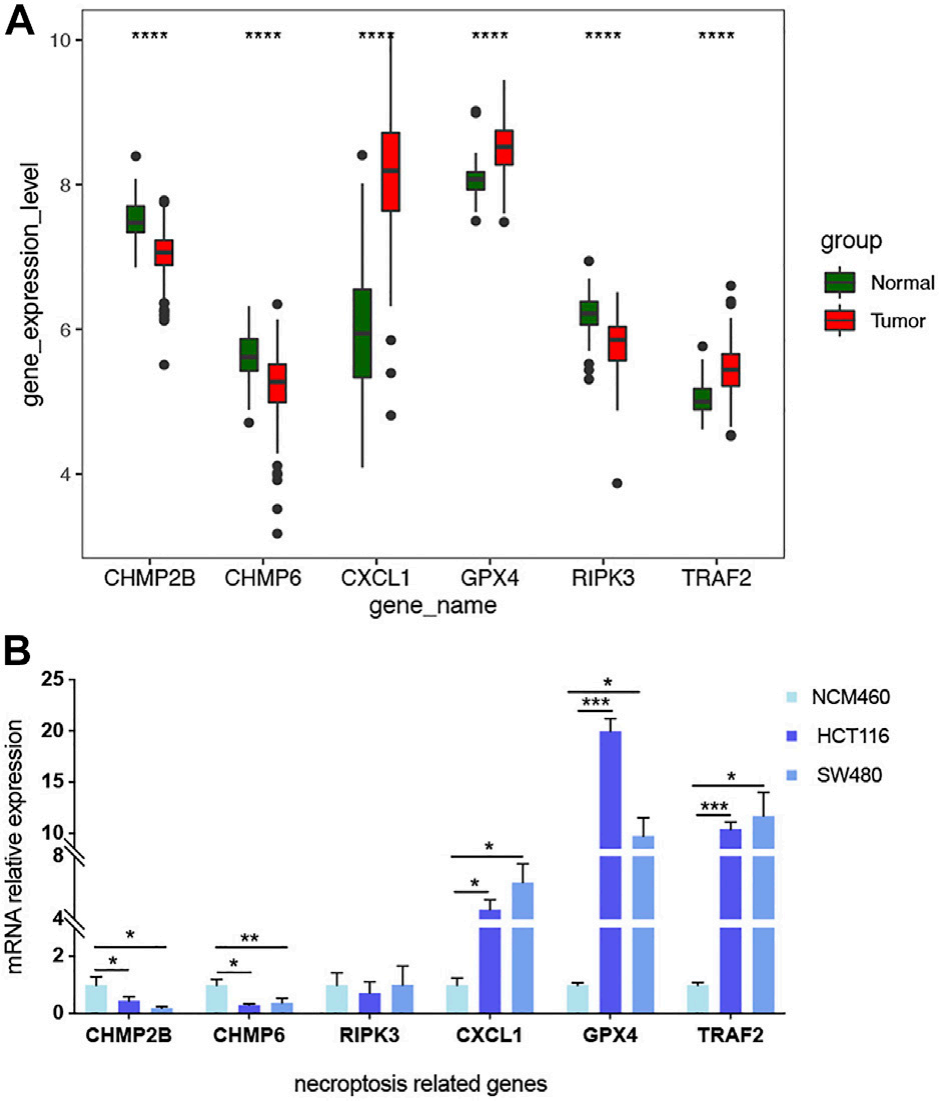
mRNA levels of 6 prognostic signature genes validated in GSE41258 and cell lines.

The differential mRNA levels of the prognostic signature genes in the expression validation set GSE41258 are shown in Figure 10A. CHMP2B, CHMP6, and RIPK3 were downregulated, while CXCL1, GPX4, and TRAF2 were upregulated in tumor samples. We also validated the mRNA relative expression level of these genes in one normal colorectal cell line, NCM460, and two colorectal cancer cell lines, HCT116 and SW480 (Figure 10B). We found that the mRNA levels of these genes are consistent with the above results based on the expression validation set GSE41258, except RIPK3. The mRNA expression levels of CHMP2B and CHMP6 were lower, while CXCL1, GPX4, and TRAF2 showed higher mRNA expression levels in HCT116 and SW480 than NCM460. However, the result of RIPK3 showed no significance.

## Discussion

Necroptosis is a programmed cell necrosis that is mainly activated by receptor-interacting protein kinase 1 (RIPK1), and mediated by receptor-interacting protein kinase 3 (RIPK3) and mixed lineage kinase domain-like pseudokinase (MLKL) (Shan et al., 2018). It is involved in the pathological process of various diseases including tumors (Strilic et al., 2016). A recent study determined two necroptosis-related clusters and a prognostic signature including 10 genes based on bioinformatic analysis, making risk stratification and treatment optimized in hepatocellular carcinoma (Chen et al., 2022). Necroptosis was also found to promote the progression of Stanford type A aortic dissection (TAAD) by activating immune infiltration (Liu et al., 2022).

Colorectal cancer is one of the most common malignant tumors with high incidence and mortality, which is still a major threat to global health. The pathogenesis and progression of tumors are closely concerned with necroptosis, and the vulnerability to necrosis is also considered to be a potential weakness of malignant tumors, which may be targeted for antitumor therapy (Gong et al., 2019). However, the role of NRGs in CRC is still unclear with few studies.

Our study built a prognostic signature on the basis of DNRGs in CRC and obtained six biomarkers, including CHMP2B, TRAF2, RIPK3, CXCL1, GPX4, and CHMP6. Previous research has demonstrated that TRAF2 acts as both a negative regulator of death receptor-induced apoptosis and an inhibitor of TRAIL- and CD95L-induced necroptosis (Karl et al., 2014). RIPK3 is a serine-threonine protein kinase that is a key regulator of infection-induced necroptosis, which plays different roles in different types of tumors (Liu et al., 2021). It has been proven that CXCL1-promoted macrophage-induced adaptive immunosuppression can be induced by necroptosis to advance the occurrence of pancreatic ductal adenocarcinoma (Seifert et al., 2016). There are few studies on the association of necroptosis with CHMP2B, GPX4, and CHMP6.

In our study, the low-risk group was enriched by several KEGG pathways, such as nod-like receptor signaling pathway, toll-like receptor signaling pathway, cytokine receptor interaction, cytosolic DNA sensing pathway, and p53 signaling pathway. Among them, toll-like receptors (TLRs) have been proven to play an important role *via* evolutionary-conserved motifs for recognizing the pathogen-associated molecular patterns (PAMPs) in CRC (Medzhitov, 2001; Akira and Takeda, 2004; Shibolet and Podolsky, 2007). When the toll-like receptor signaling pathway is dysregulated, it may lead to an imbalance of intestinal epithelial cells (IECs) and the development of CRC (Moradi-Marjaneh et al., 2018). Furthermore, previous studies have confirmed that chemokines and chemokine receptors can promote CRC metastasis, and their abnormal expression may contribute to the prognosis of CRC (Mitchell et al., 2019). P53 is a frequently mutated tumor suppressor gene in CRC that promotes the growth of cancer and leads to treatment resistance (Liebl and Hofmann, 2021). The p35 signaling pathway was mediated by Cullin-4B to promote cell proliferation and invasion in CRC (Zhong et al., 2021), and was thought to be a potential targeted signaling pathway for the treatment of CRC (Slattery et al., 2019). In addition, immune microenvironment analysis demonstrated that 32 immune checkpoints were significantly different between high- and low-risk groups, such as ADORA2A, BTNL2, and CD160. ADORA2A is an adenosine receptor, and blocking tumor-produced adenosine perhaps enhances the effect of immunotherapy (Raskovalova et al., 2007). BTNL2 is considered an effective inhibitor of the antitumor immune response, which has been confirmed in mouse models (du et al., 2022). The high mRNA level of CD160 in CRC has been confirmed in several studies (Saleh et al., 2020; Ma et al., 2021).

According to drug sensitivity analysis based on GDSC, patients in the high-risk group were found to be more sensitive to Lapatinib. Lapatinib is an inhibitor of human epidermal growth factor receptor type 2 (HER2) and epidermal growth factor receptor (EGFR), which is applied in combination with capecitabine in the treatment of HER2-positive metastatic breast cancer (Geyer et al., 2006). Lapatinib monotherapy or Lapatinib combined with trastuzumab can benefit patients with HER2-positive metastatic CRC to some extent (Guan et al., 2020; Tosi et al., 2020). In contrast, patients in the low-risk group showed more sensitivity to lenalidomide. Lenalidomide is usually used in combination with dexamethasone for the treatment of relapsed or refractory multiple myeloma (Dimopoulos et al., 2007). A study in 2013 indicated that lenalidomide significantly activated T cells, and its combination with cetuximab significantly enhanced the immune regulatory effect of KRAS-mutant metastatic CRC (Gandhi et al., 2013). Although it was confirmed that lenalidomide can reduce tumor vascular density in a mouse model of CRC with liver metastasis, the antitumor effect of lenalidomide in solid tumors still requires further study (Leuci et al., 2016).

Our study further completes the field of the role of necroptosis in CRC to some degree. At present, the research works on necroptosis in CRC mainly concentrate on certain substances or specific signaling pathways, while there are few in-depth discussions on NRGs in colorectal cancer. For example, the resistance of hypoxic colorectal cancer cells to necroptosis is produced by the glycolytic metabolism, partly *via* scavenging mitochondrial free radicals (Huang et al., 2013). In addition, RIP3-mediated necroptosis forms an important part of Resibufogenin’s suppressing growth and metastasis of CRC (Han et al., 2018). Some substances like fragile X mental retardation protein (FMRP), ABIN-1, PFK-15, and carnosine can regulate necroptosis in CRC in different ways. FMRP regulates necroptosis through the surveillance of the RIPK1 mRNA metabolism (di Grazia et al., 2021). ABIN-1 is a key regulator, and its deficiency may facilitate necroptosis-based antitumor therapy by increasing the sensitivity of CRC cells to necroptosis (Cai et al., 2021). The cytotoxicity and genotoxicity of PFK-15 are attenuated if necroptosis is suppressed in CRC cells (Yan et al., 2021). Carnosine can suppress CRC cells’ proliferation *via* the *ß*-catenin/Tcf-4 signaling pathway, thereby inducing necroptosis, and is considered a potential compound from diet for the treatment and prevention of CRC (Hsieh et al., 2022). The abovementioned studies integrate a complete map of the further mechanisms underlying the role of necroptosis in CRC. Recently, one research has provided a more comprehensive analysis of NRGs in colon cancer (CC) based on bioinformatics (He et al., 2022). It involves prognosis, immune infiltration, and drug sensitivity analysis but mainly focuses on cluster analysis of the necroptosis molecular subtypes in CC. Nevertheless, this study has some imperfections compared with ours in terms of both the range of disease and the depth of analysis. In addition to immune microenvironment infiltration analysis and drug sensitivity analysis, we constructed a prognostic signature and screened out 6 significant related biomarker genes. Of course, our study still has some shortcomings and limits, such as the small sample size, retrospective study using only bioinformatics methods to obtain data from public databases, and lack of diversity in the type and number of samples for *in vitro* validation experiments. As a result, although we used ROC curves, line plots, and gene expression experiments for validation, further and deeper validation experiments are still needed. In addition, gene RIPK3 was not validated at the level of cell lines, which may be ascribed to the limitations of cell lines. Despite the lack of further experiments to confirm our findings, it is undeniable that our study lays the foundation in the field of necroptosis, and we will continuously pay attention to the DENRGs.

## Conclusion

Our study established a six-gene signature comprising CHMP2B, TRAF2, RIPK3, CXCL1, GPX4, and CHMP6, and a prognostic nomogram, which can reliably predict the overall survival of CRC. In addition, we provide a range of clinical risk factors for patients with CRC available for reference.

## Data Availability

The datasets presented in this study can be found in online repositories. The names of the repository/repositories and accession number(s) can be found below: https://www.ncbi.nlm.nih.gov/geo/, GSE39582 https://www.ncbi.nlm.nih.gov/geo/, GSE41258.
